# Lipid-lowering drugs in ischaemic heart disease: A quasi-experimental uncontrolled before-and-after study of the effectiveness of clinical practice guidelines

**DOI:** 10.1186/1471-2261-11-47

**Published:** 2011-08-04

**Authors:** Rosa Dalmau, Miriam Boira, Carina Aguilar, Carlos López, Dolors Rodríguez, Delicia Gentille, Domingo Bofill, Eduard Diogene, Josep M Pepió

**Affiliations:** 1 ABS Tortosa-Est, Institut Català de la Salut (ICS), Institut Universitari d'Investigació en Atenció Primària (IDIAP) Jordi Gol, Tortosa, Spain; 2 ABS Flix, Institut Català de la Salut (ICS), Institut Universitari d'Investigació en Atenció Primària (IDIAP) Jordi Gol, Flix, Spain; 3 ABS Tortosa-Oest, Institut Català de la Salut (ICS), Institut Universitari d'Investigació en Atenció Primària (IDIAP) Jordi Gol, Tortosa, Spain; 4 Unitat de Suport a la Recerca Terres de l'Ebre, Institut Universitari d'Investigació en Atenció Primària (IDIAP) Jordi Gol & IISPV, Tortosa, Spain; 5 Fundació Institut Català de Farmacologia, Universitat Autònoma de Barcelona, Spain; 6 Internal Medicine and Cardiology Departaments, Hospital Verge de la Cinta, Institut Català de la Salut (ICS) & IISPV, Tortosa, Spain

## Abstract

**Background:**

Cardiovascular diseases(CVD), specifically ischaemic heart disease(IHD), are the main causes of death in industrialized countries. Statins are not usually prescribed in the most appropriate way. To ensure the correct prescription of these drugs, it is necessary to develop, disseminate and implement clinical practice guidelines(CPGs), and subsequently evaluate them.

The main objective of this study is to evaluate the effectiveness of the implementation of consensual Lipid-lowering drugs (LLD) prescription guidelines in hospital and primary care settings, to improve the control of Low-Density Lipoprotein Cholesterol (LDL-C) levels in patients with IHD in the Terres de l'Ebre region covered by the Catalonian Health Institute. Secondary objectives are to assess the improvement of the prescription profile of these LLDs, to assess cardiovascular morbimortality and the professional profile and participant centre characteristics that govern the control of LDL-C.

**Methods/Design:**

Design: Quasi-experimental uncontrolled before and after study. The intervention consists of the delivery of training strategies for guideline implementation (classroom clinical sessions and on-line courses) aimed at primary care and hospital physicians. The improvement in the control of LDL-C levels in the 3,402 patients with IHD in our territory is then assessed.

Scope: Primary care physicians from 11 basic health areas(BHAs) and two hospital services (internal medicine and cardiology).

Sample: 3,402 patients registered with IHD in the database of the Catalan Institute of Health(E-cap) before December 2008 and patients newly diagnosed during 2009-2010.

Variables: Percentage of patients achieving good control of LDL-C, measured in milligrams per decilitre. The aim of the intervention is to achieve levels of LDL-C < 100 mg/dl in patients with IHD. Secondary variables measure type and time of diagnosis of IHD, type and dose of prescribed cholesterol-lowering drugs, level of physician participation in training activities and their professional profile.

**Discussion:**

The development of prescription guidelines previously agreed by various medical specialists involved in treating IHD patients have usually improved drug prescription. The guideline presented in this study aims to improve the control of LDL-C by training physicians through presential and on-line courses on the dissemination of this guideline, and by providing feedback on their personal results a year after this training intervention.

## Background

CVD, specifically IHD, is the leading cause of death in Europe overall, and specifically in Spain. It is also responsible for high morbidity and high economic health care costs. Therefore, it is of particular concern in the health plans in industrialized countries [1]. At present, CVD prevention is based on the detection and treatment of modifiable risk factors, including dyslipidaemia [1-4].

For DL, lipid-lowering treatment aims not only to reduce levels of the different blood lipids, but also to reduce the CV morbidity and mortality associated with them. It is considered that the value of LDL-C should determine the start of lipid-lowering therapy since there is strong evidence that such a reduction leads to a directly proportional reduction in CV risk [2,5]. Thus, hypercholesterolaemia is accompanied by a high prevalence of coronary disease in both sexes, explaining 19.4% of coronary events in men and 20.1% in women. The information currently available about the risk attributable to each of the cardiovascular risk factors (CVRFs) in the Spanish population is limited. Although we know the prevalence of these CVRFs we do not know very much about their control [6].

In order to improve the management of CVD and IHD various organizations have developed a number of CPGs and Expert Consensus Documents in recent years. These documents review the scientific evidence related to IHD, CVD CVRFs and CV prevention, critically assess the diagnostic and therapeutic procedures, and establish recommendations based on the profile of efficacy, safety, convenience and cost of a procedure or treatment, and assess the risk/benefit [1-3,5,7]. After developing these CPGs it is imperative to translate the knowledge they yield to professionals and thereby to clinical practice. Unfortunately, it is well known that professionals who should be using these CPGs are often unaware of them or simply do not implement them [8-10]. This is why it is necessary to develop strategies for CPG implementation that can overcome the difficulties and barriers in the setting in which they are going to be used. These strategies have had a favourable influence on clinical outcomes. It is also essential to make a final analysis of the results using polls and registers to verify whether physicians are implementing the recommendations of the guidelines in their clinical practice and to assess their impact on patients' clinical outcomes [8-10].

Some studies have aimed to determine the theoretical applicability of the GPCs by comparing results of clinical practice in the field of the DL and primary care. The RESPECT study [11] has shown moderate levels of agreement between theoretical calculations and routine clinical practice. Several clinical trials, in which the intervention consisted of the dissemination of guidelines for hypertension and DL, have reported an increase in the prescription of drugs, but no improvement in the quality of the management of hypertension and DL patients [11,12]. Another study of CPG implementation in primary care and CVD has shown the quality of care to be suboptimal. In this study, only 15-40% of patients achieved the treatment goals. Medical education interventions and subsequent audits offer improvements but there remains a gap between the recommendations and the reality [13].

In the health region of Terres de l'Ebre, there is particular interest in and concern to improve the management of patients with CVD. The Preseap study has recently been carried out in our area [14]. This aimed to improve the management of patients in secondary prevention of CVD, through a programme of intensive control based on medical visits by patients in their health centre. The results showed no differences between this group of patients and a control group who made regular visits [14,15]. In addition, the factors determining the control of LDL-C levels have been studied in patients with CVD. It was found that control of these factors differed within and between the various studies, an outcome that should be borne in mind when applying the current recommendations for achieving therapeutic goals in secondary prevention [16].

For this reason, a collaborative action between primary and hospital care units was instigated in 2008 to coordinate, harmonise and improve the management of patients with CVD in our area. Amongst these actions, a multidisciplinary CPG for the treatment of DL in secondary prevention of CVD has been planned, with technical advice from the Catalan Institute Pharmacology Foundation (FICF).

In this CPG, very general recommendations are made about the control of various CVRFs, and the non-pharmacological (lifestyle changes) and pharmacological DL treatments (especially hypercholesterolaemia) are also described in detail. In addition, the efficacy, safety, convenience, cost and risk-benefit ratio of various therapeutic interventions and their role in secondary prevention are evaluated. It also provides several tables for guidance to facilitate drug prescription by physicians based on the goals of LDL-C control for secondary prevention. Finally, an algorithm is proposed to improve patient control and follow-up.

In recent years, the need to promote research into primary care has become increasingly recognised, as exemplified by the many studies addressing the development of strategies to improve prescription quality and implementation of GPCs [10]. One of the great challenges that faces us at present is to ensure that all the professional involved adhere closely to the GPCs [7,17-20].

## Objectives

The main objective is to evaluate the implementation effectiveness of a set of lipid-lowering treatment guidelines in the primary care and hospital settings, and thereby to improve LDL-C lipid control in patients with IHD throughout the Terres de l'Ebre Region served by the Catalonian Institute of Health.

### Secondary objectives

- To evaluate the implementation effectiveness of the guidelines for improving the profile (type and dose) of LLD prescribed by physicians to patients with IHD.

- To assess CV morbidity and mortality.

- To determine how professional profile influences the impact and effectiveness of the intervention.

## Methods/Design

### Study design

The design is a quasi-experimental uncontrolled before-and-after study, investigating patients seen by physicians in primary care centres and hospitals in the Terres de l'Ebre region covered by the Catalan Institute of Health.

### Setting

All the Primary Care Areas (PCAs) of the Terres de l'Ebre provincial Health Region of Tarragona, Catalonia, Spain, have agreed to participate in this study. This will include physicians from the eleven PCAs and cardiologists and internal medicine physicians from the Hospital de Tortosa Verge de la Cinta.

### Study period

The study will be undertaken between January 2009 and January 2012.

### Study sample

The sample will include those patients registered in the database (e-cap programme) with different IHD-related diagnoses: myocardial infarction, stable angina or unstable coronary syndrome, and chronic myocardial ischaemia. In all, 3,402 patients (registered up to December 2008) will participate in this study, along with those newly diagnosed with IHD who have been registered in the e-cap programme during 2009 and 2010. All patients who do not meet the criteria of the attended population (i.e., those not making a minimum of three primary care visits over the previous two years, and at least one visit in the last year) will be excluded from the study. Patients with a terminal or serious illness will also be excluded.

### Intervention

The intervention has been designed to use different strategies for implementing the CPG in an attempt to increase the efficiency of lipid-lowering prescription for better control of DL in the patients in our health region. The intervention will be carried out by medical professionals through double training sessions with a brief refresher session of the GPC and provision of feedback of their personal results a year after the initial intervention.

The intervention targeted at the physician has two components

#### a) Presential training sessions and interactive workshops

Doctors from the 11 primary care centres and the two hospital services (Cardiology and Internal Medicine) will be informed about the CPG and the various measures of secondary coronary heart disease prevention (how to achieve the target levels of LDL-C). Two doctors (one from primary care and the other from a hospital), previously trained by the research team, will lead the sessions that will take place in each primary care centre and hospital service. Two types of session will be held:

- An initial two-hour training session.

- A one-hour refresher session, a year after the initial training session, where the initial results will be presented and attendees will be reminded of the key points.

#### b) On-line training course

On a voluntary basis, physicians involved in the study may take a 20-hour on-line course. Teachers will be physicians from the FICF.

### Measurements

First, a database will be set up to contain the cases diagnosed with IHD, wherein every case will be linked to the primary care physician and health centre. This will allow us to correlate the patient's degree of disease control with their sociodemographic characteristics, doctor's profile and primary care centre characteristics. We will analyze the number of patients with LDL cholesterol control (patients with LDL cholesterol < 100 mg/dl), mean of controlled patients (with LDL cholesterol < 100 mg/dl) per physician, number of patients with IHD by physician, types of IHD and years of disease progression. Patients with and without lipid-lowering therapy, treatment type, dosage and proper treatment of IHD (numbers of patients with: anti-aggregation vs. anticoagulation, beta blockers and angiotensin-converting enzyme inhibitors (ACEI)) will also be analysed.

The number of laboratory tests per patient before and after the intervention will be taken into account to assess the degree of patient control.

The profile of the clinicians who receive the intervention will include variables such as their attendance on the classroom course, completion of the on-line course, their socio-demographic characteristics, employment status, specialist by residency training system or not, and assistance quality standards (scored from 0 - 1000 on the basis of the recommendations of an expert group. This number is determined by the level of detection and control of the most prevalent diseases), standard of drug prescription (scored from 0 - 130 according to the annual prescription standards set by an expert panel). Finally, characteristics of each primary care centre, such as population assignment, population served, number of patients per doctor, status as a teaching or non-teaching centre, location in a rural or urban area, and whether a referral hospital, will also be examined.

#### Main independent variable

Percentage of patients who achieve a good control of LDL-C, measured in milligrams/decilitre (mg/dl). The control goal for IHD patients is LDL-c < 100 mg/dl.

At the beginning of the study, a patient will be considered to be controlled if the most recent measured level of LDL-C (in 2008) is < 100 mg/dl.

#### Secondary variables

##### IHD

- Type: Acute myocardial infarction, stable and unstable angina, acute coronary syndrome and chronic myocardial ischaemia.

- Time since disease diagnosis (diagnosis date)

##### LLD

- Type of the LLD(s) prescribed.

- Doses of the LLD(s) prescribed.

#### Number of laboratory tests of LDL-C

- Number of laboratory tests of LDL-C performed before (in 2009), during (2010-2011) and after the intervention (in 2012) in patients with an IHD diagnosis.

#### Assessment of professional participation

- Number of physicians who have attended the presential (classroom) session.

- Number of physicians who have undertaken the on-line course.

#### Sociodemographic characteristics of physicians

- Hospital or primary care physician, gender, age, employment status (steady or locum), specialist by residency training system or not, Catalan Health Institute referral hospital or not, teaching unit, assistance quality standards and drug prescription standard.

#### Characteristics of Primary Care Centres

Drug prescription quality standard, assistance quality standards, assigned population, attended population, number of patients by doctor, teaching or non-teaching primary attention centre, rural or urban location, and referral hospital status.

### Statistical analysis

#### 1. Sample size

At present, the percentage of good control of LDL-C levels of patients with IHD in our area is around 35% (assistance quality standards results for our area calculated from the primary care information services of Catalonia Institute of Health). Considering this value as a starting point, we estimate that it would be reasonable to expect an increase in control efficiency of at least 10% following this intervention.

We assume an average of 25 patients per physician with a 95% confidence interval (CI) of 0.05. Accepting an alpha error of 0.05 and 90% statistical power for bilateral contrasts and assuming a 20% loss rate, would require an estimated sample size of 2,761 patients. To achieve this sample, given a 2.4% prevalence of IHD in our population of 142,549 adult inhabitants, 115,042 health service users will be needed.

#### 2. Analytical strategy

The statistical analysis will be preceded by an examination of outliers, data cleaning, identification and labelling of missing and/or unenforceable values, and labelling of variables and variable categories.

Variables will be summarised as the mean and 95% CI for continuous variables, and as absolute values or percentages and 95% CI for categorical variables. Statistical significance will be concluded for values of p < 0.05. The difference in percentage control of LDL-C levels between baseline and the end of the study will be assessed using the McNemar test. To assess the improvement of control of c-LDL an analysis of covariance using logistic regression will be carried out. Subsequently, a multilevel logistic regression model will be developed. The main independent variable in the both models will be "intervention", adjusted for baseline values and potential confounders. Physician will be considered a cluster in the multilevel regression).

### Ethical aspects

The presented project has been evaluated by the Clinical Research Ethics Committee of the primary care research institute, IDIAP Jordi Gol, Barcelona June 12, 2009 (Ref: P09/25). Since the project examines specific information about the illness control and the information was obtained anonymously from a population approach (before and after intervention), the Committee did not consider necessary to require the Informed Consent. The intervention was applied to all patients and there were not neither comparative groups nor randomized distribution.

The study guarantees the confidentiality of professionals' data. The data collected and generated will be included in two databases as a data decoupling procedure to adhere to data protection legislation and to maintain individual privacy. For this reason there will be different types of files, each with a different password to be renewed periodically and which shall only be accessible by a responsible person.

The first file will be a demographic database, in which each patient is assigned a unique code. The second file will include the patient codes linked to the main clinical variables relevant to the study that will include the results of the study and the clinical variables for statistical analysis. The databases will remain stored on a hard disk of a PC with a renewable password located in a locked office with controlled access.

### Forecast execution dates

The project will be carried out over three years.

September 2009: presentation of training material, standardized training for trainers.

January 2010: classroom and face-to-face satisfaction survey.

March 2010-June 2010: on-line course.

January 2011-March 2011: post-intervention analysis of results.

May 2011 Feed-back and reminder of GPC.

January 2012-September 2012: data processing, statistical analysis, data interpretation, preparation and dissemination of results.

## Discussion

The limitations of the project are that it is a before-and-after intervention study without a control group, randomisation or masking. No control group has been proposed in order to ensure that the educational intervention reaches the maximum number of professionals.

Presential training courses are being undertaken by both physicians in hospitals (cardiologists and internists) and primary care settings, since both groups are responsible for the diagnosis and monitoring of patients with IHD.

In fact, all the 11 PCAs have two referral laboratories for the determination of the LDL-C but both offer the same quality guarantees for this value determination. Another limitation may be that the follow-up period is insufficiently long to detect changes in morbidity and mortality and prescription habits of physicians. However, we hope to detect at least a trend towards an improvement in our results.

Previous studies of the implementation of guidelines do not show very encouraging results regarding the overall improvement of the quality of patient care but do indicate an increase in drug prescription [11,12].

This project is interesting because vascular disease has a high morbidity and mortality. In the Spanish population as a whole, this has been the leading cause of death for many decades. It causes severe disability among the population and is responsible for a substantial increase in healthcare costs.

Statins are the drugs of first choice for the treatment of DL in patients with IHD and have been shown to reduce cardiovascular morbidity and mortality. They are also easy to use and have a good clinical safety profile.

Despite the evidence of the need to achieve a LDL-C level of ≤ 100 mg/dl for patients with IHD, this occurs in only 35% of patients in our area (2008 data). The collaboration between primary care and hospital units with the help of the aforementioned methodology seeks to achieve a degree of control sufficient to decrease morbidity and mortality among these patients, as well as to clear the way for the development of consensual guidelines to unify patient management criteria and significantly improve the health of our population.

## List of abbreviations

CVD: cardiovascular disease; IHD: Ischaemic heart disease; CPG: clinical practice guideline; LLD: Lipid-lowering drugs; LDL-C: low-density lipoprotein-cholesterol; BHA: Basic health areas; CVRF: cardiovascular risk factor; PCA: primary care area; CI: confidence interval.

## Competing interests

The authors declare that they have no competing interests.

## Authors' contributions

RD is the principal investigator and has participated in the conception of the project and the study design, drafting of the manuscript, in the intervention with the physicians and in the analysis and dissemination of the results. MB and CL have participated in drafting this manuscript. MB, CA and JMP have contributed to the project concept and study design. MB and DB have participated in the intervention courses. DR has managed the on-line course. CA has carried out the statistical analyses. JMP, DB, DG and ED have participated in drafting the CPG. All authors have read all the different versions and approved the final version of this manuscript.

## Pre-publication history

The pre-publication history for this paper can be accessed here:

http://www.biomedcentral.com/1471-2261/11/47/prepub
